# Validation and reliability of the Turkish version of the life transition scale for parents of children with disabilities: a methodological study

**DOI:** 10.1186/s12887-026-06544-9

**Published:** 2026-02-09

**Authors:** Kamile Akça, Soner Berşe

**Affiliations:** 1 Faculty of Health Sciences, Gaziantep Islam Science and Technology University, Gaziantep, Türkiye; 2 Faculty of Health Sciences, Gaziantep University, Gaziantep, Türkiye

**Keywords:** Children with disabilities, Parents, Physical and intellectual disability, Reliability and validity, Scale

## Abstract

**Background:**

Parents of children with disabilities often experience significant psychosocial challenges during the process of accepting and adapting to their child’s condition. To better understand and support this life transition, valid and reliable instruments are needed. This study aimed to evaluate the validity and reliability of the Life Transition Scale for parents of disabled children within the Turkish context.

**Methods:**

This methodological study was conducted with 154 parents of children with physical or intellectual disabilities attending two special education and rehabilitation centers in eastern of Türkiye. The scale was adapted through translation, back-translation, expert review, and pilot testing. Content validity was assessed using the Davis technique. Explanatory factor analysis and confirmatory factor analysis were performed to assess construct validity. Internal consistency was evaluated using Cronbach’s alpha. Additional analyses included item-total correlations, independent t-tests, and ANOVA for group comparisons.

**Results:**

Explanatory factor analysis revealed four factors—Wandering, Denying, Accepting, and Despairing—explaining 65.5% of the total variance. Factor loadings ranged from 0.619 to 0.830. Confirmatory factor analysis supported the model with acceptable fit indices. The Cronbach’s alpha coefficients were 0.925 for Wandering, 0.889 for Denying, 0.858 for Accepting, 0.843 for Despairing, and 0.935 for the overall scale. Item-total correlations ranged from 0.369 to 0.728, all statistically significant (*p* < 0.001).

**Conclusions:**

The Turkish version of the Life Transition Scale is a valid and reliable instrument for assessing the life transition experiences of parents of children with disabilities. This scale can inform evidence-based family-centered interventions and policy development to support parental adaptation and well-being.

**Supplementary Information:**

The online version contains supplementary material available at 10.1186/s12887-026-06544-9.

## Introduction

Caring for a child with a disability is a complex, multifaceted process that significantly impacts the emotional, social, and physical well-being of parents. The World Health Organization (2023) estimates that approximately 16% of the global population lives with some form of disability, underscoring the widespread relevance of this issue [1]. Among caregivers, parents—particularly mothers—of children with disabilities experience unique psychological burdens due to continuous caregiving responsibilities, role shifts, and chronic uncertainty about their child’s development and future [2–4].

Globally, informal caregiving for children with disabilities imposes substantial emotional and physical strain, particularly in low- and middle-income countries where access to support services is limited. Studies from diverse cultural contexts—including Nigeria, Switzerland, Uganda, and Korea—have consistently documented high caregiver burden, social isolation, and reduced quality of life among parents of children with disabilities [5–8]. In Türkiye, similar findings have been reported, with elevated caregiver burden directly linked to diminished quality of life among parents of children with chronic or developmental conditions [9]. This universal yet culturally mediated burden underscores the need for cross-cultural tools and interventions that are both evidence-based and contextually relevant.

The caregiving experience often disrupts normative life trajectories, producing high levels of stress, fatigue, and social isolation. Empirical studies consistently reveal elevated rates of depression, anxiety, and emotional exhaustion among these parents, compounded by limited access to formal support and persistent societal stigma [2, 10]. Moreover, the caregiving burden is not static—it evolves over time and is shaped by the interplay between parents’ coping strategies, resource availability, and the severity of the child’s condition [3, 11].

In psychometric research, the development and validation of instruments must be guided by a sound conceptual framework to ensure construct clarity and contextual relevance. Conceptual grounding informs item selection, highlights the rationale behind the theoretical domains, and strengthens the interpretability of scale outputs [12]. For instance, the development of caregiver-related instruments such as the CARe Burn Scale emphasized theory-informed domains and stakeholder input to achieve valid representation of caregiver experience [13]. Without such theoretical anchoring, psychometric tools risk measurement bias, poor cultural fit, and limited generalizability, particularly in cross-cultural adaptation studies. Therefore, aligning item content with established theoretical constructs—such as transition theory in caregiving—is critical to ensure both content validity and cross-cultural appropriateness.

To understand the dynamic nature of parental adaptation, transition theory offers a robust conceptual framework. Meleis’s Transitions Theory posits that major life events—such as assuming lifelong caregiving for a disabled child—unfold through identifiable phases involving disruption, disorganization, and eventual integration [11]. In the context of caregiving, transition theory has been operationalized in the Life Transition Scale (LTS), which was originally developed and later validated in separate studies conducted in different caregiver populations [14, 15]. The LTS delineates four emotional stages in the caregiving experience: denying, wandering, despairing, and accepting. These stages reflect the evolving emotional responses and identity realignments parents undergo in adapting to their child’s disability.

Despite its potential, the Life Transition Scale (LTS) has undergone validation in only a limited number of cultural contexts—primarily Korea and East Asia [14, 15]. No studies to date have adapted or psychometrically validated the LTS within the Turkish caregiving population. Moreover, recent global reviews emphasize the lack of validated psychosocial assessment instruments in non-Western and middle-income contexts, warning that reliance on unadapted measures risks overlooking key cultural nuances [16, 17]. Addressing this gap is crucial for ensuring equitable inclusion of culturally diverse caregiving populations in nursing and pediatric research. Given the psychosocial risks associated with caregiving and the utility of transitions theory in conceptualizing parental adaptation, there is a pressing need for a culturally adapted and validated instrument that can reliably assess the caregiving transition process. Such a tool would inform clinical interventions, guide policy on family support services, and enrich the global discourse on disability caregiving.

In light of these gaps, this study aims to fill a critical void by evaluating the psychometric properties—specifically the validity and reliability—of the Life Transition Scale among Turkish parents of children with disabilities. By conducting a culturally sensitive adaptation and validation, this research provides a tool that reflects the unique emotional trajectories of caregiving in Türkiye while offering comparative insights that contribute to the global literature on disability caregiving.

## Methods

### Design and setting

This methodological study was conducted between July 2022 and April 2023 at two special education and rehabilitation centers in eastern of Türkiye, both affiliated with the Provincial Directorate of National Education. These centers provide multidisciplinary rehabilitation services (special education, physical therapy, and psychological counseling) to children with physical, intellectual, and developmental disabilities. The institutions were selected for their accessibility and diverse service populations, ensuring inclusion of parents from various sociodemographic and disability backgrounds. In Türkiye, families obtain eligibility for special education through the Guidance and Research Center, which issues an official disability report following multidisciplinary evaluation. Children aged 1–18 with verified disability reports are accepted into these centers for standardized educational and rehabilitation support.

### Population and sample

Participants were parents of children with officially documented intellectual or physical disabilities enrolled in the selected centers. Convenience sampling was used. Inclusion criteria were:

- being the primary caregiver of a child with an officially verified disability;
- sufficient literacy to complete questionnaires;
- absence of psychiatric or cognitive impairment; and
- voluntary participation.

Sample size was determined using the recommended ratio of 5–10 participants per scale item. For the 24-item Life Transition Scale, this required 120–240 participants. A total of 154 parents were recruited (≈ 6.5 participants per item), satisfying methodological standards for factor analysis. Although some literature suggests larger samples for confirmatory factor analysis, contemporary evidence emphasizes that adequacy depends on model complexity and factor loadings rather than fixed numeric thresholds [18]. Thus, the sample size was deemed sufficient for stable factor and reliability analyses.

### Instruments

The data of the study were collected using the “Descriptive Information Form” created by the researchers, and the “The Life Transition Scale”.

#### Descriptive information form

The descriptive information form was prepared by the researchers, and consisted of the characteristics of parents and children, including questions such as the age of the parent, education level, the age of the child, and the type of disability of the child.

#### The life transition scale (LTS)

This scale was developed by Lee et al. [14] in order to examine the life transition process of parents of children with autism. Later, Hong et al. [15] tested its validity and reliability among parents of children with various types of disabilities. The scale is a four-point Likert-type instrument consisting of four subscales—Denying, Wandering, Despairing, and Accepting—and 24 items. Each item is scored from 1 (“not true”) to 4 (“completely/always true”), and subscale scores are calculated as the mean of their items (range 1–4). Higher values indicate stronger endorsement of that phase—for instance, higher Despairing reflects greater hopelessness and exhaustion, whereas higher Accepting denotes greater adaptation. Consistent with the original scale, diagnostic cut-off values are not established; therefore, the Turkish version is intended for research and clinical screening to identify relative differences and guide supportive interventions (e.g., psychoeducation or counselling for elevated Denying/Wandering/Despairing, resilience-focused support for higher Accepting).

### Procedures

In the present study, the LTS was administered in person as a self-report, questionnaire during routine center visits, and participants completed it in approximately 15–20 min. A researcher was available to clarify procedural questions but did not provide any interpretive guidance. Written permission for cultural adaptation and validation was obtained from the scale developers. The Life Transition Scale was originally developed based on Meleis’s Transitions Theory and was first validated among parents of children with autism through exploratory and confirmatory factor analyses, which confirmed a four-factor structure [14]. The stability of this four-factor model was later confirmed in a large-sample validation conducted across different disability types [15]. Internal consistency was adequate (Cronbach’s α = 0.84 for the total scale; 0.80–0.90 across subscales). The Cronbach’s alpha coefficient of the original scale was 0.84, and in the present study it was 0.935, indicating excellent internal consistency. To ensure that the translated form accurately measured the intended constructs, conceptual and semantic equivalence were maintained through forward- and back-translation, expert review, and pilot cognitive debriefing without altering item meaning.

**Fig. 1:**
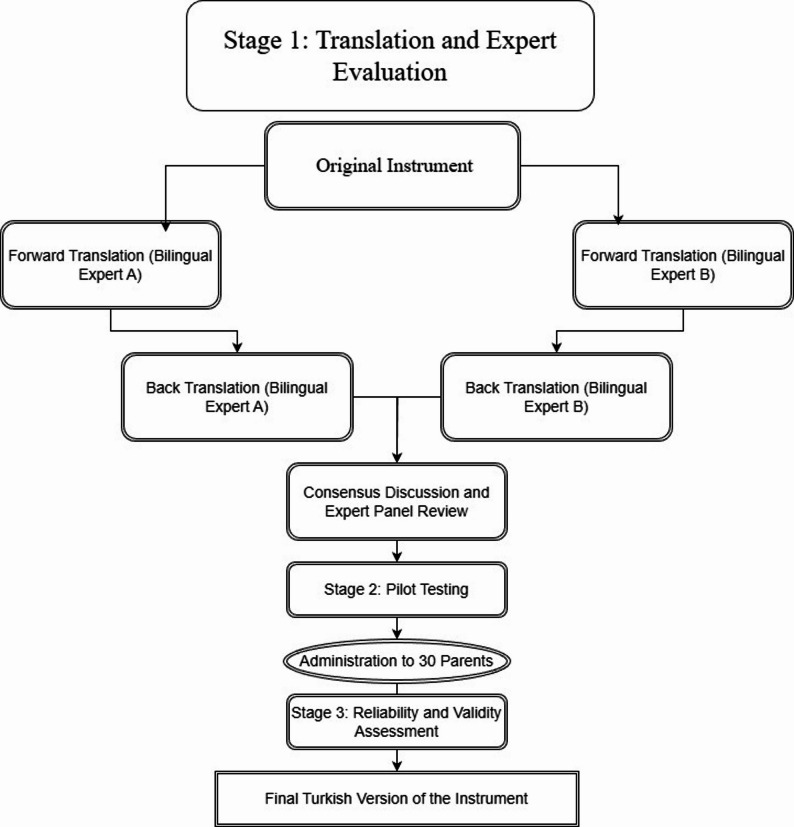
Translation and validation process of the Turkish version of the life transition scale

The translation and cultural adaptation process was conducted to ensure that the translated items retained the semantic and conceptual equivalence of the original English–Korean constructs while reflecting culturally embedded caregiving realities in Türkiye. Although the LTS has been validated in East Asian contexts, direct linguistic translation would not fully capture culturally specific emotional expressions such as shame, devotion, or resilience, which differ across collectivist societies. Therefore, a culturally sensitive adaptation was undertaken to confirm that the Turkish version appropriately represented these psychological transitions. The scale was independently translated into Turkish by two certified bilingual experts with experience in health sciences. Subsequently, two different language experts performed back-translation into English. Discrepancies were resolved through consensus discussions (Fig. 1).

Expert review was conducted by five faculty members specializing in nursing, special education, and psychometrics to ensure cultural relevance and conceptual equivalence. Prior to the main analysis, the content validity of the Turkish version of the Life Transition Scale was evaluated by a multidisciplinary panel of five experts in pediatric nursing, psychological counseling, and special education. Each expert rated the relevance, clarity, and cultural appropriateness of the 24 items using a four-point scale (1 = not relevant, 4 = highly relevant). In this study, the Davis technique was employed to assess the content validity of the scale. Content validity indicates that the scale comprehensively represents the intended concept and includes appropriate items [19]. This process is a critical step in ensuring the content validity of the scale and confirms its appropriateness and validity for the target audience. Expert consensus indicated that no item required substantial modification, only minor wording adjustments for idiomatic clarity, confirming conceptual equivalence between the Turkish and original versions.

To assess the clarity of the scale’s statements, the scale was administered as a pilot study to 30 parents with similar characteristics to the sample group. As a result of the feedback received from the parents, any unclear parts of the scale were revised to ensure clarity and comprehensibility. Furthermore, individuals included in the pilot application were not incorporated into the sample.

### Data analysis

All statistical analyses were conducted using IBM SPSS Statistics version 25 and AMOS version 21. Prior to the main analyses, data were screened for missing values, outliers, and assumptions of normality. Skewness and kurtosis values were examined, with thresholds of ± 1.5 considered acceptable for normal distribution. Descriptive statistics were calculated for demographic variables and scale items, including frequencies, percentages, means, and standard deviations.

To examine the construct validity of the Life Transition Scale, both Exploratory Factor Analysis (EFA) and Confirmatory Factor Analysis (CFA) were employed. The principal component analysis with Varimax rotation was used to identify the factor structure. The Kaiser–Meyer–Olkin (KMO) measure of sampling adequacy and Bartlett’s test of sphericity were applied to assess data suitability for factor analysis. Following EFA, CFA was performed to confirm whether the identified four-factor model provided a satisfactory representation of the data in the Turkish context. Internal consistency reliability was assessed using Cronbach’s alpha coefficient, with a value of ≥ 0.70 considered acceptable. Additionally, item-total correlations and Cronbach’s alpha if item deleted were calculated to evaluate the contribution of individual items. The eigenvalues, explained variance, and factor loadings were also reported.

To further assess group differences, independent samples t-tests and one-way ANOVA were conducted based on sociodemographic characteristics such as parental education level, age, and child’s type of disability. A significance level of *p* < 0.05 was adopted for all inferential statistics.

### Ethical considerations

In order to conduct the research, firstly, approval (Meeting Date: March 23, 2022; Decision Number: 2022/49) from the Clinical Research Ethics Committee of Gaziantep University was obtained, and institutional permission was obtained from the centers where the research would be conducted (Document ID: 99957001/2022-14). Permission was also obtained from the author of the original scale (Hwal Lan Bang), via e-mail for validity and reliability analysis. In addition, written informed consent was obtained from the parents who agreed to participate in the study. In this study adhered to the Helsinki Declaration of Human Rights.

## Results

Before performing factor analysis, the assumptions of sample adequacy and data suitability were tested. The Kaiser–Meyer–Olkin (KMO) measure of sampling adequacy was 0.887, indicating that the sample size was *perfectly sufficient* for factor analysis [20]. Bartlett’s Test of Sphericity was significant (χ²(276) = 2358.326; *p* < 0.001), confirming that the correlation matrix was appropriate for factor extraction.

Item-Level Content Validity Index (I-CVI) values ranged from 0.83 to 1.00, exceeding the recommended threshold of 0.78. The Scale-Level CVI/Average (S-CVI/Ave) was 0.94, and the Universal Agreement Index (S-CVI/UA) was 0.88, indicating excellent consensus. Inter-rater agreement measured by Cohen’s kappa (κ = 0.86) demonstrated substantial reliability of expert evaluations. Qualitative feedback confirmed that the Turkish items accurately reflected culturally embedded emotional expressions such as parental guilt, social stigma, and acceptance phases.

### Exploratory factor analysis

To explore the factor structure of the Turkish LTS, an Exploratory Factor Analysis was conducted using Principal Component Analysis with Varimax rotation. Although the original LTS proposed a four-factor model, EFA was performed to verify whether the same construct structure held in the Turkish cultural context.

The analysis revealed four factors with eigenvalues greater than 1, collectively explaining 65.5% of the total variance. Factor loadings ranged from 0.619 to 0.830, demonstrating strong item representation within each factor. The variance explained by each factor was as follows:

- Wandering: 21.8%
- Denying: 16.2%
- Accepting: 15.7%
- Despairing: 11.8%

These findings confirm that the Turkish version of the LTS retained the original four-factor structure, with all items demonstrating adequate factor loadings and conceptual alignment (Table 1).

**Table 1:** Explanatory factor analysis and reliability results of the life transition scale for parents of disabled children

Factors and items	Factor Loading	Explained Variance (%)
Wandering (α = 0.925)		21.8
Item 7	0.778	
Item 12	0.769	
Item 8	0.747	
Item 13	0.745	
Item 10	0.720	
Item 9	0.706	
Item 14	0.700	
Item 11	0.679	
Denying (α = 0.889)		16.2
Item 6	0.756	
Item 1	0.754	
Item 5	0.723	
Item 4	0.715	
Item 3	0.697	
Item 2	0.653	
Accepting (α = 0.858)		15.7
Item 17	0.813	
Item 18	0.803	
Item 16	0.716	
Item 20	0.713	
Item 19	0.688	
Item 15	0.674	
Despairing (α = 0.843)		11.8
Item 22	0.830	
Item 24	0.796	
Item 23	0.763	
Item 21	0.619	
Total (α = 0.935)		65.5

KMO = 0.887; χ2(276) = 2358.326; Bartlett Test of Sphericity (p) = 0.00

Table 2 shows the independent group t-test results showing the discriminating power of all items and item-total correlation. The minimum value required for the item-total test correlation to be sufficient is specified as 0.30 [21]. The item-total test correlation values ​​of the answers given by the participants to the scale questions were examined, and it was determined that there were no items below 0.30. The item-total test correlation values ​​of the remaining items varied between 0.369 and 0.728. As seen in the item-total test correlation table, it was determined that all remaining items were related to each other. In order to determine the distinctiveness of the items in the scale, the raw scores obtained from the scale were ordered from largest to smallest. The mean scores of the high 27% and low 27% groups were compared with the independent group t-test. As a result of the comparison, it was seen that there was a statistically significant difference between the averages of the low and high group item scores. From this point of view, it can be stated that the scale is distinctive in terms of measuring the desired quality.

**Table 2:** Item analysis of the subscales of the ‘Life transition scale for parents of disabled children’ for participants (*n* = 154)

Item Number	Item Total Score Correlation	(High % 27-Low %27) t	(Low % 27-High %27) *p* value
Item 7	0.653	−8.772	< 0.001
Item 12	0.696	−10.571	< 0.001
Item 8	0.715	−13.218	< 0.001
Item 13	0.728	−9.546	< 0.001
Item 10	0.708	−10.164	< 0.001
Item 9	0.716	−12.181	< 0.001
Item 14	0.689	−8.509	< 0.001
Item 11	0.617	−11.054	< 0.001
Item 6	0.633	−7.542	< 0.001
Item 1	0.561	−4.680	< 0.001
Item 5	0.554	−6.822	< 0.001
Item 4	0.656	−7.619	< 0.001
Item 3	0.658	−5.906	< 0.001
Item 2	0.631	−8.919	< 0.001
Item 17	0.505	−0.129	< 0.001
Item 18	0.495	−0.219	< 0.001
Item 16	0.439	−0.590	< 0.001
Item 20	0.481	−1.345	< 0.001
Item 19	0.479	−1.263	< 0.001
Item 15	0.369	0.000	< 0.001
Item 22	0.466	−6.761	< 0.001
Item 24	0.528	−8.195	< 0.001
Item 23	0.571	−7.793	< 0.001
Item 21	0.644	−7.236	< 0.001

**Fig. 2:**
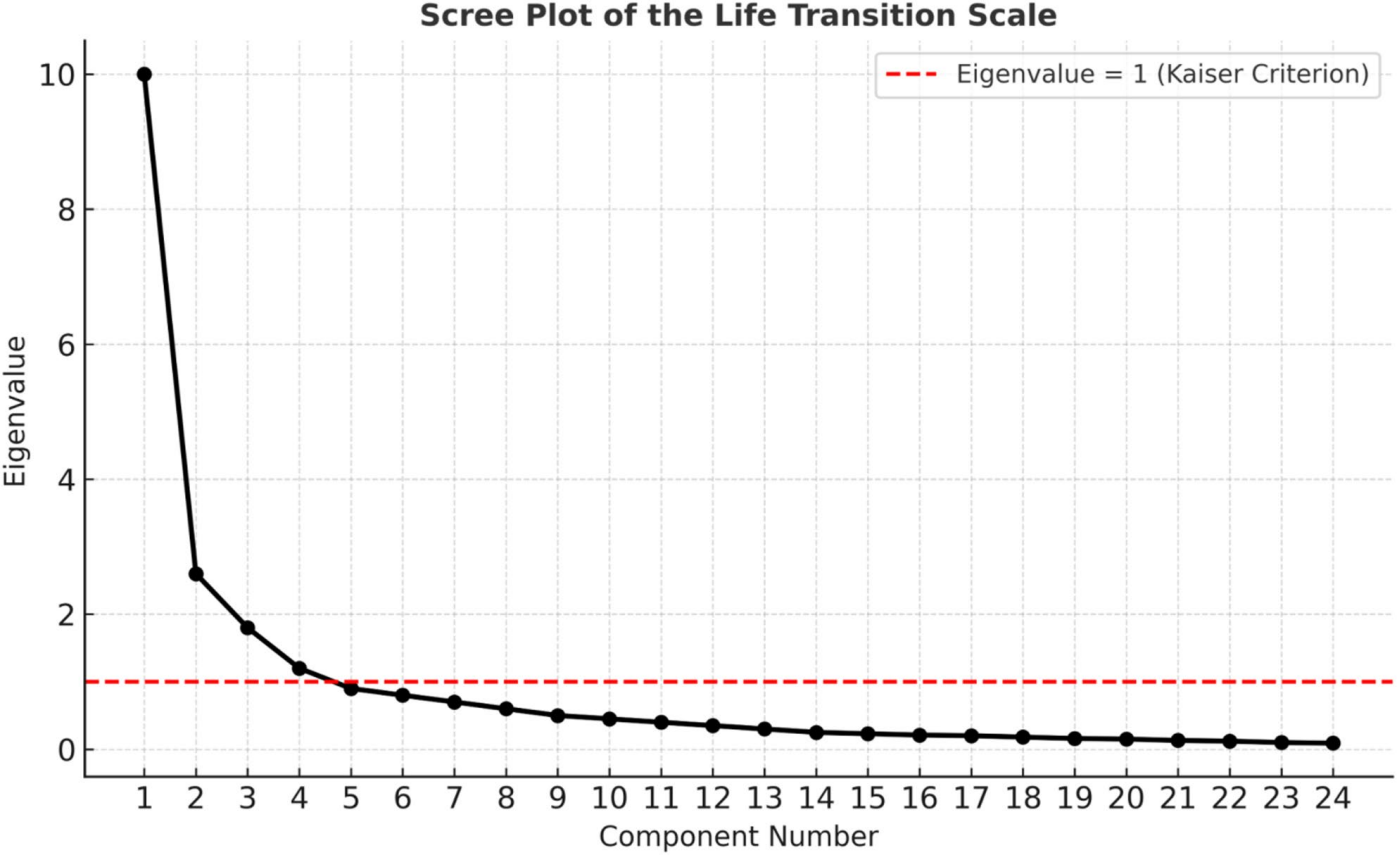
Scree plot of the life transition scale for parents of disabled children

When the graph, which includes the number of factors on the horizontal axis and the eigenvalues on the vertical axis, is examined, it is seen that the high-accelerated fall decreases after the fourth point. The trend of the declines seen from the first point shows the degree of contribution to the variance. After the fourth point, the contribution of each factor to the variance decreases, and it is seen that the contributions of the variances to be added are very close to each other It was decided that there should be 4 factors in the scale according to the eigenvalue in the scree plot and percentages of variance obtained in line with the explanatory factor analyses (Fig. 2).

### Confirmatory factor analysis

Confirmatory Factor Analysis was conducted to evaluate the model fit of the Turkish version of the Life Transition Scale. As shown in Table 3, the model demonstrated acceptable goodness-of-fit (χ²/df = 1.828, RMSEA = 0.074, CFI = 0.910, TLI = 0.897, IFI = 0.911). These results indicate that the four-factor structure of the Turkish version of the scale provides a satisfactory representation of the data and supports its structural validity.

**Table 3:** Results of multi-factor confirmatory factor fit indices for the life transition scale for parents of disabled children

Index	Accepted Value	Before Modification	After Modification
χ²/df	< 5	2.018	1.828
CFI	> 0.90	0.916	0.910
TLI	> 0.90	0.874	0.897
IFI	> 0.90	0.888	0.911
RMSEA	< 0.08	0.082	0.074

It was determined that 55.8% of the participants consisted of fathers, that 53.9% were 39 years old or younger, that 86.4% were married, that 53.9% had high school or higher education, and that 68.8% had low income. It was found that 45.5% of the participants had a child with a physical disability and that 46.8% had a child aged 6 years or younger (Table 4).

**Table 4:** Investigation of the sociodemographic differences

			Denying		Wandering		Despairing		Accepting	
Variables	Categories	*n* (%)	Mean ± SD	t or F (*p*)	Mean ± SD	t or F (*p*)	Mean ± SD	t or F (*p*)	Mean ± SD	t or F (*p*)
Parent Age	≤ 39^a^	83 (53.9)	14.59 ± 2.65	9.529 (0.000*)	15.84 ± 3.56	16.554 (0.000*)	7.96 ± 2.13	10.973 (0.000*)	15.57 ± 2.12	10.697 (0.000*)
	40− 49^b^	43 (27.9)	15.67 ± 3.50		17.90 ± 4.90		8.81 ± 3.23		15.76 ± 3.72	
	≥ 50^c^	28 (18.2)	17.64 ± 4.19		21.07 ± 4.85		10.67 ± 3.07		12.64 ± 4.33	
Post hoc analysis				c> a		c> a		c> a		a> c
				c> b		c> b		c> b		b> c
						b> a				
Marital Status	Married	133 (86.4)	15.24 ± 3.39	1.923 (0.056)	17.20 ± 4.63	1.129 (0.261)	8.54 ± 2.77	1.706 (0.090)	14.97 ± 3.42	1.135 (0.258)
	Single	21 (13.6)	16.76 ± 3.19		18.42 ± 4.53		9.66 ± 3.05		15.85 ± 2.26	
Parent	Father	86 (55.8)	15.57 ± 3.65	0.406 (0.611)	17.26 ± 4.74	0.251 (0.852)	9.02 ± 3.11	1.309 (0.101)	14.72 ± 3.40	1.261 (0.756)
	Mother	68 (44.2)	15.34 ± 3.20		17.45 ± 4.55		8.43 ± 2.56		15.39 ± 3.21	
Education	≤ Middle School	71 (46.1)	16.54 ± 3.50	3.886 (0.000*)	18.78 ± 4.90	3.657 (0.000*)	9.21 ± 3.10	2.121 (0.000*)	14.90 ± 3.86	0.680 (0.498)
	≥High School	83 (53.9)	15.34 ± 3.20		16.15 ± 4.02		8.25 ± 2.49		15.26 ± 2.74	
Income status	Low	106 (68.8)	15.74 ± 3.42	1.621 (0.107)	17.56 ± 4.79	0.780 (0.416)	8.83 ± 3.01	1.035 (0.303)	14.78 ± 3.41	1.863 (0.65)
	Middle	48 (31.2)	14.79 ± 3.28		16.93 ± 4.24		8.37 ± 2.35		15.79 ± 2.96	
Type of Disability	Intellectual	39 (25.3)	14.58 ± 3.25	1.967 (0.143)	17.12 ± 4.58	0.750 (0.928)	8.79 ± 2.96	0.168 (0.846)	14.66 ± 4.21	1.118 (0.330)
	Physical	70 (45.5)	15.92 ± 3.12		17.48 ± 4.06		8.77 ± 2.53		14.95 ± 2.38	
	Both Physical and Intellectual	45 (29.2)	15.44 ± 3.84		17.40 ± 5.51		8.48 ± 3.17		15.68 ± 3.62	
Child Age	≤ 6^a^	72 (46.8)	14.20 ± 2.43	24.220 (0.000*)	16.18 ± 3.79	16.104 (0.000*)	8.02 ± 2.28	12.307 (0.000*)	14.87 ± 2.32	7.307 (0.001*)
	7− 12^b^	51 (33.1)	15.25 ± 3.39		16.72 ± 4.27		8.77 ± 2.53		16.31 ± 2.68	
	≥ 13^c^	31 (20.1)	18.64 ± 4.42		18.64 ± 4.42		8.48 ± 3.17		13.61 ± 5.09	
Post hoc analysis				c> a		c> a		c> a		b> a
				c> b		c> b		b> c		b> c
				a> b						

*SD* Standard Deviation, *t* Independent Samples T-Test, *F* One-way analysis of variance (ANOVA)

Superscript letters (a, b, c) indicate significant differences between groups according to post-hoc test (*p* < 0.05)

^*^p < 0.05

A one-way analysis of variance (ANOVA) was conducted to examine differences in the Life Transition Scale subscale scores according to demographic characteristics. Statistically significant differences were observed among parental age groups across all four subscales—Denying, Wandering, Accepting, and Despairing (*p* < 0.05). Post hoc Bonferroni comparisons indicated that parents aged 50 years and above had significantly higher mean scores in the *Wandering*, *Denying*, and *Despairing* subscales, and lower scores in *Accepting* compared to younger parents. A significant association was also found between the child’s age and LTS subscales (*p* < 0.05). As children’s age increased, parental scores on the *Denying* and *Wandering* subscales increased, while scores on the *Accepting* subscale decreased. Specifically, parents of children aged ≥ 13 years scored significantly higher on the *Wandering* subscale compared to those with children aged ≤ 6 years. Regarding parental education, significant differences were found in the *Wandering*, *Denying*, and *Despairing* subscales (*p* < 0.05), with lower education levels associated with higher scores. No statistically significant differences were observed in the *Accepting* subscale. Furthermore, income status, parent gender, and type of child disability showed no significant relationships with any LTS subscales (*p* > 0.05) (Table 4).

Descriptive statistics were calculated for the total LTS score and its four subscales. Higher scores indicate a more advanced phase of life transition. The mean subscale scores were as follows: Wandering (M = 2.58, SD = 0.50), Denying (M = 2.42, SD = 0.59), Accepting (M = 2.47, SD = 0.53), and Despairing (M = 2.84, SD = 0.70). These results provide an overview of the participants’ transition phases prior to factor and group analyses.

## Discussion

In this study, the construct validity and reliability of the Turkish version of the Life Transition Scale (LTS) were rigorously confirmed (Table 1), aligning well with previous validations [14, 15]. Factor loadings (0.619–0.830) and Cronbach’s alpha coefficients (0.843–0.935) (Table 1) indicate strong item homogeneity and internal consistency, confirming that the Turkish version of the LTS reliably measures the underlying constructs among parents of children with disabilities in Türkiye. The findings of this study confirm that the Turkish version of the Life Transition Scale maintains a clear four-factor structure, comprising Denying, Wandering, Despairing, and Accepting, in accordance with Meleis’s Transitions Theory. This theory conceptualizes adaptation as a multidimensional process that involves psychological, social, and role redefinition phases [22]. The consistency of these domains across different cultures indicates that emotional responses to life transitions are not confined to a single society but represent universal patterns of human adaptation. Similar multidimensional structures have been reported in studies that applied Meleis’s framework to postpartum adjustment [23] and chronic illness coping [24], supporting the stability of these constructs across diverse contexts.

Compared with previous studies, the present validation shows that Turkish parents of children with disabilities experience a similar emotional trajectory, progressing from denial and uncertainty to gradual acceptance. However, in our sample, parents of older children exhibited lower scores on the Accepting subscale, suggesting that prolonged caregiving may reactivate emotional strain or diminish acceptance over time. This pattern reflects the non-linear nature of transition processes described by Meleis’s Transitions Theory, where adaptation may fluctuate across life stages [25]. Importantly, while previous studies emphasized cognitive adaptation among Korean caregivers [14, 15], the Turkish data add evidence of affective and cultural adaptation, highlighting the interaction between emotional endurance and sociocultural coping styles such as collective family support and religious meaning-making [26]. These results suggest that the theoretical structure of the Life Transition Scale is cross-culturally stable, but the experiential expression of transitions differs by sociocultural context. Similar findings from studies in Iran and Spain indicate that, although psychometric structures remain consistent, the emotional depth and significance of constructs such as denial and acceptance vary across cultures [27, 28]. Overall, these parallels confirm that the Turkish version of the scale maintains theoretical integrity while incorporating culturally grounded dimensions of emotional adaptation.

According to the reliability analysis calculations of the study, the Cronbach Alpha value for the Wandering subscale of the scale was 0.925, 0.889 for the Denying subscale, 0.858 for the Accepting subscale, and 0.843 for the Despairing subscale. The Cronbach Alpha value for the entire scale was found to be 0.935. In the study, in which families of children with autism were used as the sample group, the internal consistency of the Life Transition Scale was calculated as 0.83 [14] while he internal consistency rate of the Life Transition Scale, which is generally applied to families of children with disabilities, was found to be higher with 0.84 [15]. According to these findings, it can be pointed out that the scale, which was originally developed for families of children with autism, can be used for families of all children with disabilities in terms of reliability.

Beyond the Davis technique, extended validity indices were computed to strengthen the psychometric evidence base. The universal agreement index (S-CVI/UA = 0.88) and scale-average content validity index (S-CVI/Ave = 0.94) indicate strong expert consensus. Expert proportion of agreement averaged 0.91 across all items, and Cohen’s Kappa (κ = 0.86) confirmed substantial inter-rater reliability, exceeding international thresholds for cross-cultural validation [29]. These additional indices offer a nuanced understanding of expert agreement, thereby reinforcing the content validity and statistical credibility of the Turkish LTS.

The analysis showed no significant differences in Life Transition Scale subscale scores according to the type of the child’s disability (*p* > 0.05). Although it was expected that disability type might influence the life transition experiences of parents, this finding aligns with previous studies reporting no significant associations between disability type and family adaptation processes [15, 30]. However, other studies have indicated that parents of children with severe or complex disabilities, such as autism or intellectual impairment, may experience higher levels of stress and lower well-being due to challenges in communication and attachment [31–33]. These mixed results suggest that while the nature of the disability may influence the intensity of parental stress, the overall transition process, characterized by phases of denial, despair, and eventual acceptance, appears to occur across disability types.

The Turkish adaptation of the Life Transition Scale demanded rigorous attention to linguistic and cultural nuances to ensure conceptual equivalence. Emotional constructs such as *despairing* and *wandering* lacked direct Turkish counterparts, requiring iterative translation and cognitive interviews to capture the intended meaning. This process followed internationally recognized adaptation standards emphasizing semantic, experiential, and conceptual validity [34, 35]. Turkish caregivers frequently expressed *acceptance* through faith-based coping, family interdependence, and collective resilience—patterns that contrast with the more individual coping styles observed in Korean samples [36] and align with other Turkish psychometric adaptations emphasizing spirituality and relational identity in emotional processing [37]. These cultural refinements enhanced the instrument’s construct validity and ensured that the Turkish version authentically represents the sociocultural realities of caregiving, confirming that while the theoretical structure of the LTS is globally stable, its emotional interpretation is culturally embedded.

In a previous study, it was reported that mothers had significantly higher scores than fathers in the wandering phase [15]. In study conducted on families of autistic children, it was revealed that the wandering subscale scores of mothers were higher than those of fathers [14]. In our study, however, no statistically significant relationship was found between the parent gender variable and the subscales of the scale. It can be thought that this situation is related to that the lives of the mothers and fathers participating in the study were equally affected.

In a previous study, a significant relationship was identified only for the Accepting subscale, where parents aged 39 and under obtained higher scores in this dimension [15]. In our study, a statistically significant relationship was found between the parental age and the subscales of the scale (*p* < 0.05). The Accepting subscale score was found to be lower in parents aged 50 and over. The Despairing, Denying and Wandering subscales were found to be higher in parents aged 50 and over. From this point of view, it can be interpreted that individuals aged 50 and over accepted the child’s disability, but still showed resistance in terms of hopelessness, denial and wandering.

The coexistence of acceptance and resistance observed among parents aged 50 and over reflects the complex emotional ambivalence that often accompanies prolonged caregiving transitions. As highlighted in transition-focused research on older caregivers, acceptance of a child’s disability frequently coexists with unresolved anxiety regarding the child’s long-term welfare, caregiving continuity, and future dependency [38, 39]. Meleis’s Transition Theory posits that such non-linear emotional fluctuations are characteristic of adaptive processes, where individuals oscillate between stability and vulnerability during sustained caregiving roles [25]. Older caregivers, having experienced repeated cycles of adjustment, often reach cognitive acceptance yet remain emotionally unsettled—a dual state influenced by perceived loss of control and age-related caregiving fatigue [40]. In the Turkish sociocultural context, this ambivalence may be further intensified by collective caregiving expectations and limited institutional care options, reinforcing feelings of enduring responsibility despite emotional readiness to accept.

Parents with lower educational attainment exhibited higher Wandering, Denying, and Despairing scores, consistent with prior findings that education enhances coping, resource access, and acceptance [30]. The absence of income-related differences in the Turkish sample may reflect the strong informal kinship support networks typical of collectivist societies, which buffer financial hardship effects [8]. This finding underscores the need for culturally grounded interpretations of sociodemographic variables in psychometric research.

In our study, no significant relationship was found between the income status of the parents and the subscales. It was revealed that parents with low income levels got higher scores on the wandering, denying and despairing subscales, but got the lowest score on the accepting subscale [15]. It was also found that families of children with intellectual disability have lower incomes [41]. We interpret the reason for this finding as the fact that parents have less opportunity to work because children with intellectual disabilities need more and continuous care. In this study, although it was seen that the income status of the parents of the children with disabilities was not effective in the life transitions of the parents, it can be stated that the results of other studies affect the income level of the families of the children with intellectual disabilities worse [41].

This study offers original and critical evidence by validating the Life Transition Scale within a Turkish environment—one of the few rigorous psychometric investigations of caregiver transition processes in a middle-income, collectivist society. The adaptation shows that although the emotional trajectories of denial, wandering, despairing and acceptance may hold across cultures, they are shaped by local cultural beliefs, family systems, and gender norms [42]. Moreover, in low‐ and middle‐income settings, caregivers face distinct structural and sociocultural pressures, underscoring the need for culturally fit measurement and intervention tools [43]. By providing a validated instrument in this context, the study fills the gap in non‐Western psychometric research and supports policymakers and practitioners in identifying high-risk caregiver groups, tailoring family-centred support programmes, and integrating culturally responsive strategies within community and policy frameworks [44].

### Limitations

This study has certain limitations that should be considered when interpreting the findings. First, the data collection tools were based solely on parents’ self-reported information, which may have introduced subjective bias. Second, although efforts were made to reach a representative sample, the final sample size remained relatively limited. This constraint was primarily due to the inherent difficulties in accessing parents of children with disabilities, who often face time constraints, caregiving burdens, and logistical barriers that hinder research participation. Moreover, the study was conducted in only two special education and rehabilitation centers within a single province, which may limit the generalizability of the findings to broader populations or different regional contexts. Finally, the study did not assess temporal stability through a test–retest procedure. Future research should re-administer the scale within a short interval to evaluate measurement consistency over time, which would further strengthen the reliability evidence for the Turkish LTS.

## Conclusion

This study validated and culturally adapted the Turkish version of the Life Transition Scale for parents of children with disabilities, confirming that it is a theoretically grounded and psychometrically sound instrument for assessing caregivers’ emotional transitions. Rather than focusing solely on statistical indices, the findings reveal a coherent pattern of adaptation that progresses from denial and uncertainty toward acceptance, reflecting both universal and culture-specific dimensions of caregiving experiences.

These results matter because they provide clinicians, researchers, and policymakers with an evidence-based tool to better understand how Turkish parents navigate emotional adaptation in the context of disability. The LTS can thus be used to guide targeted psychosocial interventions, inform culturally sensitive counseling strategies, and evaluate the effectiveness of support programs for families across healthcare and educational settings. Furthermore, this study contributes to global nursing and psychological research by demonstrating how Meleis’s Transitions Theory operates across cultures, highlighting the emotional and relational dimensions of caregiving often overlooked in quantitative disability research.

Future research should extend the validation of the Turkish LTS to different regions and diverse disability groups, employ longitudinal designs to assess temporal stability, and examine its predictive relationships with well-being, coping, and quality of life. Clinically, incorporating the scale into caregiver assessments could help professionals identify early signs of emotional distress and tailor interventions that strengthen resilience and adaptation. From a policy perspective, the instrument offers a framework for developing inclusive family support systems and evidence-based mental health strategies that address the needs of caregivers of children with disabilities.

## Supplementary Information

Supplementary Material 1.

## Data Availability

The datasets generated and analysed during the current study are not publicly available due [the study’s informed consent did not include public sharing of participant data] but are available from the corresponding author on reasonable request.

## Abbreviations

LTS: The Life Transition Scale
EFA: Explanatory Factor Analysis
CFA: Confirmatory Factor Analysis
KMO: Kaiser–Meyer–Olkin
M: Mean
SD: Standard Deviation
