# Supplementary material for: Validation and reliability of the Turkish version of the life transition scale for parents of children with disabilities: a methodological study
Source: BMC Pediatr. 2026 Feb 9;26:212. doi: 10.1186/s12887-026-06544-9 (PMC12983590; doi:10.1186/s12887-026-06544-9)
Supplement: Supplementary file 1 — Supplementary Material 1. [file 12887_2026_6544_MOESM1_ESM.docx]

**The Life Transition Scale for Parents of Disabled Children**

| **Orjinal Scale**  **Item Number** | **Turkish Scale**  **Item Number** | **Items** | **Factors** |
| --- | --- | --- | --- |
| Item 6 | Item 7 | I feel sorry for myself | **Wandering** |
| Item 20 | Item 12 | I'm exhausted with no hope |  |
| Item 8 | Item 8 | I want to give up my child |  |
| Item 28 | Item 13 | I'm tired of living because of my child |  |
| Item 18 | Item 10 | My life itself is meaningless |  |
| Item 16 | Item 9 | I don't have my own life |  |
| Item 29 | Item 14 | I have a lot of burden and stress |  |
| Item 19 | Item 11 | I don't care about anybody but my child |  |
| Item 22 | Item 6 | I'm ashamed to take my child around | **Denying** |
| Item 1 | Item 1 | I feel that everything is confusing |  |
| Item 17 | Item 5 | I'm afraid of people's gaze |  |
| Item 10 | Item 4 | I don't like to meet people |  |
| Item 7 | Item 3 | I sometimes shrink up in shame because of my child |  |
| Item 5 | Item 2 | I want to hide my child's disability |  |
| Item 11 | Item 17 | I feel joy in my child | **Accepting** |
| Item 23 | Item 18 | I think my child is a special gift from God |  |
| Item 9 | Item 16 | I've gained rather than lost from my child |  |
| Item 27 | Item 20 | My life was enriched because of my child |  |
| Item 24 | Item 19 | My child made me thankful for small things |  |
| Item 2 | Item 15 | I feel thankful to my child |  |
| Item 12 | Item 22 | It breaks my heart to think of my child | **Despairing** |
| Item 26 | Item 24 | I'm at a loss about the future |  |
| Item 14 | Item 23 | I'm unhappy comparing my child with others |  |
| Item 3 | Item 21 | I feel fear and hopeless |  |
